# Agricultural production structure and inequality of educational development in China

**DOI:** 10.3389/fpsyg.2022.982344

**Published:** 2022-11-10

**Authors:** Jingzhou Wei, Yawen Yu

**Affiliations:** ^1^College of Economics and Management, Southwest University, Chongqing, China; ^2^College of Finance, Anhui University of Finance and Economics, Bengbu, China

**Keywords:** agricultural main producing areas, agricultural production structure rationalization index, education Gini coefficient, residents' happiness, China

## Abstract

Unbalanced regional development in China has always been the focus of the government's attention. Agricultural development in China's main agricultural regions is characterized by relatively obvious features, which are mainly manifested in the excessive concentration of agricultural production on one crop or a few agricultural products. Whether this trend of concentration will help to improve the inequalities in China's educational development is an important question for this study. Based on China's population, education and agricultural data over the past 20 years, this paper provides an in-depth analysis of educational inequalities in five typical agricultural-producing provinces by calculating indicators such as the rationalization index of agricultural production structures, the average number of years of schooling of residents and the Gini coefficient of education, in order to analyze the essential reasons for the development of education inequality in major agricultural producing areas. The results show that the urban-rural gap is an important factor affecting the equality of educational development in the main agricultural production areas; the reduction of the rationalization index of agricultural production structure can promote the improvement of inequality in educational development and narrow the urban-rural educational development gap; it also shows that the improvement of specialization in major agricultural producing areas is conducive to reducing educational inequality in major agricultural producing provinces; these conclusions provide a useful reference for narrowing the urban-rural education gap in the main agricultural production areas.

## Introduction

The regional coordinated development strategy is one of the “five overall plans” put forward by the Third Plenary Session of the 16th Central Committee of the Communist Party of China. This strategy guides all regions to carry out economic construction based on their advantages, forming a new pattern of mutual promotion, complementary advantages and cooperative development. In Xinjiang, Heilongjiang, Henan and other provinces, due to their unique natural conditions, compared with other non-major agricultural production areas, the primary industry occupies a larger proportion of their industrial structure. In these major agricultural provinces, the development of primary industry mainly depends on the production of one or a few kinds of agricultural products. Therefore, the proportion of agricultural income in the farmer's income structure is relatively high. Taking Xinjiang as an example, in the past 10 years, the added value of agriculture, forestry, animal husbandry, and fishery in Xinjiang, the main cotton-producing area, has stabilized at about 15% of the regional GDP (CNBS).

Changes in the structure of agricultural production have a profound impact on all aspects of social development, including rural education (Doležalová et al., 2014; Tluczak, 2016). Agricultural production structure is the industrial composition, inter-industry relationship and proportional relationship formed in the process of agricultural production. Its essence is the different production patterns generated by the different allocation ratios of input of various agricultural sectors, namely, the composition of planting, forestry, animal husbandry and fishery, and their mutual connection and proportional relationship. Different agricultural production structures will produce different economic benefits, will form different types of urban-rural gaps, and will also have different effects on the unequal development of education.

Considering the importance of the agricultural economy in the main agricultural producing provinces, the impact of this structural change seems to be more pronounced in the main agricultural producing areas. The agricultural production resources in the main agricultural producing areas are often concentrated in the production of a few agricultural products. For example, Xinjiang mainly grows cotton, and Heilongjiang mainly grows soybeans. When agricultural production is excessively concentrated on one crop, it will lead to changes in the structure of agricultural production in the region. Judging from typical cases, in the past 10 years, the proportion of cotton production in Xinjiang, the main cotton-producing area, in the country has increased from 53.67% in 2011 to 87.32% in 2020, and the average proportion in the past 3 years has been above 85%. At the same time, the proportion of the rural population remains high. According to data from the seventh national census, as of the end of 2020, the rural population in Xinjiang exceeded 11.23 million, accounting for 43.47%. In the context of accelerating urbanization, more than 330 million floating population will flow to cities and towns in 2020, and the proportion of the rural population nationwide has dropped to 36.11%.

Due to the limitation of the agricultural production structure, the urbanization rate of Xinjiang is relatively slow. However, the disposable income gap between urban and rural residents is constantly widening (Yuan et al., 2020; He and Du, 2022). The per capita income gap in Xinjiang has widened from 10,072 yuan in 2011 to 20,782 yuan in 2020. At the same time, the urban-rural education gap is also widening. According to the China Statistical Yearbook, taking Xinjiang's urban and rural junior high schools as an example, the gap in the number of teachers has increased from 22,894 in 2014 to 34,164 in 2020, and the gap in the number of students has increased from 319,347 in 2014 to 423,683 in 2020.

Because of the typical urban-rural dual structure in Chinese society, this feature is particularly obvious in the main agricultural-producing areas. Therefore, the study of the equality of education development in agricultural main producing areas cannot be ignored one aspect is the difference between urban and rural education development, and the other aspect is the trend of agricultural development. In China's major agricultural producing areas, agricultural production is excessively concentrated on one or several agricultural products. Whether this concentration trend is conducive to improving the inequality of education development in China's major agricultural producing areas is very important for solving the unequal development of education in major agricultural producing areas.

Against the above background, this paper focuses on five major agricultural producing provinces in China (Xinjiang, Heilongjiang, Henan, Shandong and Guangxi) to study the impact of changes in agricultural production structure on regional education equality. The structure of this paper is as follows: the second part reviews the related research on agricultural production structure and regional education level; the third part introduces the data sources and research models; the fourth part is the empirical results and analysis; the last part is a summary of the main conclusions.

## Literature review

Over the past 30 years, a large body of research has focused on educational inequality. More than 6,300 articles can be found by searching “educational inequality” on the web of science and focusing on the classification of “educational research.” A large number of documents have recorded the interaction between education inequality and socio-economic development, race, gender differences, regional differences, institutional differences, etc. (Rumberger and Larson, 1998; Colclough et al., 2000; Magnuson et al., 2004; Reay, 2006; Boliver, 2011; Hanushek and Woessmann, 2011; Reardon, 2011; Gillborn et al., 2012; Bol and Herman, 2013; Spaull, 2013; Schmidt et al., 2015; Reardon and Portilla, 2016). From the previous scholars' research, the performance of educational inequality in different countries and regions is not the same, but there are similar performances in school differences (Gamoran, 2001; Borman and Dowling, 2010; Dawson, 2010; Owens et al., 2016), early selection of educational trajectory (Bynner and Joshi, 2002; Archer and Yamashita, 2003; Bassok et al., 2016), family differences (Van Zanten, 2005; Chudgar and Luschei, 2009; Lörz et al., 2016), and uneven distribution of educational resources (Skrtic, 1991; Chiu and Khoo, 2005; Marks et al., 2006). Previous studies on education inequality in China mainly focused on gender, population, region, policy and so on (Zhou et al., 1998; Lynch and Baker, 2005; Hannum et al., 2008).

Due to the problem of unbalanced regional development, the equality of educational development in China and even in the world has always been the focus of regional development research (Li, 2008; Fleisher et al., 2010; Yang et al., 2014; ParkHouse and Rong, 2016). A study based on educational inequality in 134 countries shows that there was a slight transitional increase in education inequality to be beneficial at a very low average level of schooling, but detrimental to growth at a relatively high average level (Sauer and Zagler, 2014). Another decomposition study based on the Gini coefficient of education in 171 countries shows that: educational inequality is decreasing over the observed sample period around the globe (Sauer, 2019). Hannum and Wang (2006) use 2000 census data on year and location of birth and educational attainment to study the shifting ties between geography and educational outcomes in the population. Based on a provincial panel dataset, Liu and Ma (2018) use a generalized Theil index to measure inequality, providing quantitative and comprehensive evidence for the development of higher education resource distribution in China's provinces.

Generally, the research on educational development is mainly divided into two aspects, namely educational achievements and educational distribution (Lee and Barro, 1993, 1996). In the existing literature on educational development performance, scholars often use “enrolment rate” as an indicator to measure the achievements of educational development. At the same time, in many empirical studies, “average years of education” is more used (Psacharopoulos and Arriagada, 1986). Foreign scholars Psacharopoulos and Arriagada believe that the average years of education is an appropriate indicator in reflecting educational achievements.

As for the study of education distribution, it mainly focuses on the differences in educational attainment among regions and whether the distribution of educational resources is equitable. In some literature, educational standard deviation (O'Neill, 1995) and educational Gini coefficient (Sheret, 1988) have become important indicators for scholars to calculate the degree of educational equity. With the deepening of research in the field of education, the Gini coefficient of education has become an important universal index to measure the degree of educational equity in the world. This important trend can be seen from the use of Chinese data and transnational data by some scholars. Based on the reality of the Chinese government's promotion of the integration of urban and rural compulsory education, some scholars' research proposes a compulsory education resource allocation balance measurement index system of two parts, which are a mandatory indicator system and a control index system, with the former being the minimum standard that must be met (Xiong et al., 2018). Some scholars studied the inequality of Japanese education, and the results showed that: (1) the degree of inequality in the distribution of education in Japan is declining overall; however, the trend toward greater equality is not occurring uniformly; (2) education is more fairly distributed for females than for males; and (3) the relationship between average years of schooling and the Gini education coefficient is an inverted-U shape (Hojo, 2009).

Based on measuring educational equity indicators, scholars further study the factors that affect educational development, trying to find the reasons for the unequal phenomenon of regional educational development. The causes of educational inequality need to be analyzed from multiple perspectives. Through the study of the intergenerational transmission of educational inequality, it is found that the distribution of financial funds in the field of education and the financing mechanism of public schools have reduced intergenerational mobility and made the transmission of educational inequality more stable between generations (Zheng and James, 2022). Research from a spatial perspective shows that the relative concentration of wealth and poverty is an important factor in educational inequality (Otero et al., 2021). A study of educational inequality in Spain confirms this view (Romero-Sanchez et al., 2020). A study of educational inequality in rural areas of southwest China finds that both local government income and rural residents ' income are positively correlated with the supply of educational resources (Guo et al., 2020). From a more micro perspective, factors such as shadow education (Ku et al., 2022), gender differences (Scheeren and Bol, 2021) and social origin (Stocké et al., 2019) can aggravate the development of educational inequality. Through the study of the relationship between education and human capital, it is found that the effect of inequality in schooling on income inequality is very low, but a positive relationship is found in the case of OECD countries (Földvári and van Leeuwen, 2011). With the attention of scholars on the development of education in China, since the 1990s, the education development and education equality of ethnic minority areas and ethnic minorities in China have received extensive attention from academia. Studies have shown that dominant ethnic groups in a society often have an advantage over ethnic minorities in educational attainment, and this advantage is stable (Weiner, 1983). This phenomenon promotes researchers to find the reasons for the differences in ethnic education results. Scholars want to analyze these reasons and develop targeted measures to promote inter-ethnic education equity (Buchmann and Hannum, 2001). Studies have shown that family background and ethnic identity are closely linked (Kao and Thompson, 2003), which shows that the differences in educational opportunities between ethnic groups are mainly derived from family background, including parents' educational level, occupation status and family income and other factors, while the rural population and urban population show significant differences in these three characteristics (Fejgin, 1995). In the study of ethnic education inequality: Researchers found that China is different from other countries, China's institutional design has an important impact on the education and status of ethnic minorities (Hannum, 2002). The research on the relationship between education and agricultural development mainly focuses on developing countries. For example, the study on the relationship between agriculture and education development in Zambia. The research shows that education has contributed to the overcoming of the personal and cultural constraints on the development of farming in Zambia (Vanzetti and Bessell, 1974).

Scholars hold different views on how to solve the problem of educational inequality. Through the study of Indian group education, scholars have found that pure education policy intervention has little effect on narrowing the education gap between different groups (Arughese and Bairagya, 2020). Another study of educational inequality in Spain shows that compulsory primary education can reduce educational inequality at the lower stages of education (Fernández-Mellizo, 2022). A study of the development of educational inequalities in South Asian countries shows that governments can reduce educational inequalities by providing free education in poor areas and developing employment schemes (Munir and Kanwal, 2020). A study of educational inequality in 34 provinces in Indonesia found that educational inequality can be reduced by improving local government education expenditures and increasing unconventional expenditures (Wirandana and Khoirunurrofik, 2022). A study of Brazil's agricultural structure and educational inequality found that more equitable land distribution can reduce the development of educational inequality (Wegenast, 2010). From a more macro perspective, the increase of education expenditure in developing countries can reduce the development of education inequality (Tan and Wang, 2021).

Although there have been many literatures on China's educational development performance and regional equity, few literatures focus on the educational development of the main agricultural producing provinces, especially the impact of special factors such as changes in agricultural production structure on educational development. Therefore, based on the population, education and agricultural data of China's five main agricultural production areas in the past 20 years, this paper calculates the level of education development, equity and rationalization index of agricultural production structure. And based on this study, the impact of changes in agricultural production structure on the development of education.

To sum up, this paper has the following three contributions: First, this paper focuses on the education development in the main agricultural production areas, filling the literature gap; second, this paper combines the Lorenz curve model to analyze the rationalization index of agricultural production Defined; thirdly, this paper studies the impact of changes in agricultural production structure on educational equity.

## Data sources and research methods

### Data source

The data used in this paper can be mainly divided into three categories. When calculating the education development index, the data includes all provinces in China from 2002 to 2019; when calculating the agricultural rationalization production index, the data includes the five major agricultural production areas from 2013 to 2018 (part of the data before 2013 is missing, and the data after 2018 unpublished); when analyzing Xinjiang as a typical case, the time frame of the data is nearly 20 years. All data are from the “China Statistical Yearbook” “China Population and Employment Yearbook” “China Agricultural Yearbook” “National Agricultural Products Cost-benefit Data Compilation,” and the 1st to 7th China Population Census Statistical Announcements.

### Research methods

In empirical studies, average years of schooling is commonly used as a measure of educational attainment (Lee and Barro, 1993). For the measurement of educational equity, the commonly used methods are the standard deviation of education (Ram, 1990) and the Gini coefficient of education (Psacharopoulos and Arriagada, 1986). Thomas et al. (2001) argues that the Gini coefficient of education is a more effective indicator of changes in the development of educational equity over time series. For the measurement of the rationalization of agricultural production structure, the rationalization index of agricultural production structure is also constructed based on the Lorenz curve model (Li et al., 2002). The main reason for using these two methods is that both methods are based on the Lorenz curve model, which can effectively express the imbalance of education and agricultural production structure.

#### Indicators of education performance and inequity

1. Calculation of average years of education. Using population data grouped by educational level, the weighted average of overall educational level is calculated with the proportion of population at all levels of education as weight. The calculation formula is:

(1) $$\begin{array}{l} \mu =\sum_{\text{i}=\text{1}}^{\text{n}}\text{pi}yi \end{array}$$

Among them, *i* represents the level of education, *i* = 1, 2, 3, 4, 5 represent five different levels of education, respectively, not attending school, primary school, junior high school, senior high school and college and above, and *n* = 5 represents the highest level of education; pi is the proportion of the population with the highest education level *i* in the total population; *yi* is the number of years with the highest level of education of *i*. Referring to the conventional processing mode, the *Yi* values of no school, primary school, junior middle school, high school and college and above are assigned to 0, 6, 9, 12, 16 years respectively.

2. Measurement of educational inequality. In the study of educational inequality, Gini coefficient is widely used, and there are many calculation formulas of Gini coefficient. The formula used in this paper to calculate the Gini coefficient of education is shown in (2).

(2) $$\begin{array}{l} G\;=\;\sum_{i=2}^{\text{n}}\sum_{j=1}^{i-1}pi\left(yi-yj\right)pj/\mu \end{array}$$

In formula (2), μ is the average years of education of the population, Pi represents the proportion of the total population of the sample population with educational level *i*, pj represents the proportion of the total population of the sample population with educational level *j, yi* represents the average educational years of the sample population with educational level *i, yj* represents the average educational years of the sample population with educational level *j, i*, and *j* represent the sample population with the highest educational level in the group.

3. Decomposition of educational Gini coefficient. Through the decomposition of educational inequality in the main agricultural producing areas between urban and rural areas, the key contradictions of educational inequality in the main agricultural producing areas can be clearly found. For this purpose, this paper adopts the method proposed by Zhang and Li (2002), and the decomposition formula is shown in Formula (3).

(3) $$\begin{array}{l} G={\text{p}}_{1}^{2}\left(\mu 1/\mu \right)G1+{\text{p}}_{2}^{2}\left(\mu 2/\mu \right)G_{2}+G_{B} \end{array}$$

Among them, μ is the average education years of the whole population, pi(*I* = 1, 2) represents the population proportion of subgroups, μ_i_ represents the average education years of subgroups, Gi represents the Gini coefficient of subgroup education, GB represents the contribution of subgroup differences to total education inequality, G represents the Gini coefficient. Thus, total educational inequality is broken down into two parts: the sum of absolute contributions of subgroup 1 and subgroup 2, and the absolute contribution of differences between subgroups. If the total educational inequality is standardized to 1, then formula (3) can be expressed as formula (4).


(4) $$\begin{array}{l} 1=\frac{{\text{p}}_{1}^{2}\left(\mu 1/\mu \right)G}{G}+\frac{{\text{p}}_{2}^{2}\left(\mu 2/\mu \right)G_{2}}{G}+\frac{G_{B}}{G} \end{array}$$

In Formula (4), $\frac{{\text{p}}_{1}^{2}\left(\mu 1/\mu \right)G}{G}$ represents the relative contribution of subgroup 1, $\frac{{\text{p}}_{2}^{2}\left(\mu 2/\mu \right)G_{2}}{G}$ represents the relative contribution of subgroup 2, and $\frac{G_{B}}{G}$ represents the relative contribution of difference between subgroups.

#### Agricultural production structure rationalization index

**Figure 1:**
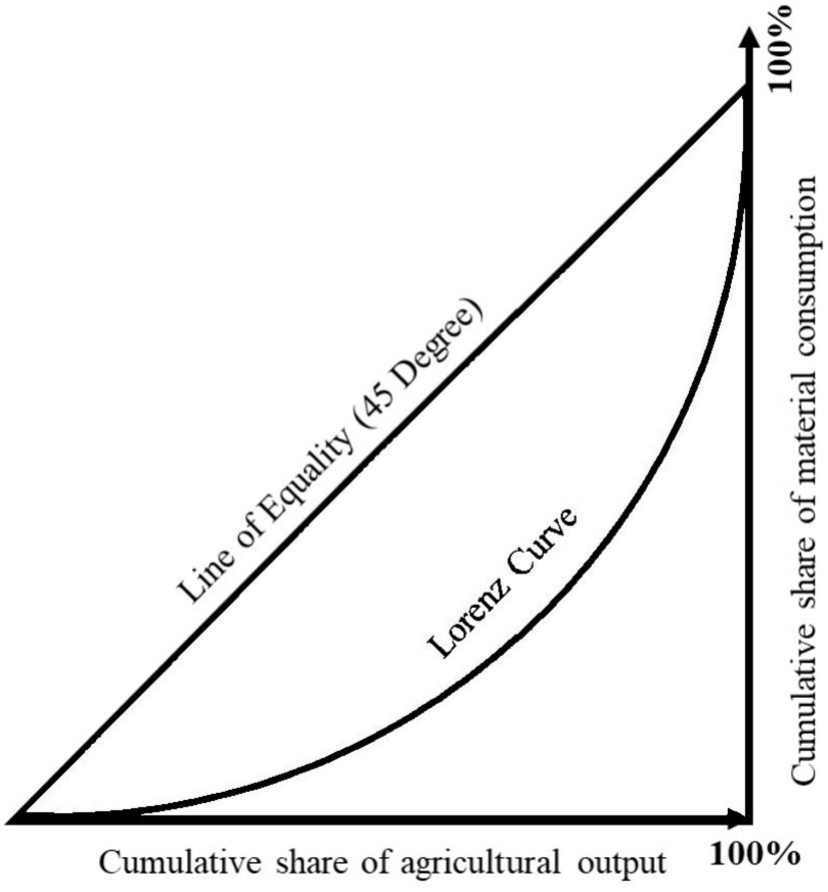
Lorentz curve model.

Lorenz curve model is applied to the research on the rationalization of agricultural production structure. The cumulative percentage of output term (i.e., agricultural output value) and the cumulative percentage of input term (i.e., material consumption) in a certain year are used to represent the horizontal axis and the vertical axis, respectively. The diagonal can be expressed as the coordinated distribution line. The area between the coordinate distribution line and the horizontal axis is divided by the area between the Lorenz curve and the horizontal axis, and the result is the rationalization index of the agricultural production structure. As shown in Figure 1 below.

The value of the final calculation results is between 0 and 1, the closer to 1, indicating that the degree of rationalization of agricultural production structure is higher, the closer to 0, indicating that the rationalization index of agricultural production structure is lower. In the actual calculation process, the input-output coefficient of each department (the ratio of the output value of each department to the material consumption of each department) is calculated, and the departments are sorted according to the order from high to low. Then the cumulative percentage of output value and material consumption are calculated respectively. Finally, the part between Lorenz curve and transverse axis is approximated as composed of several curved trapezoids by mathematical method, and the area of trapezoid is used for approximate calculation. The calculation formula is shown in Equation (5).


(5) $$\begin{array}{l} RT=\overset{n}{\underset{i=1}{\sum }}\frac{\left(Y_{i-1}+Y_{i}\right)\times \left(X_{i}-X_{i-1}\right)}{100\times 100} \end{array}$$

In Equation (5), RT is the rationalization index of agricultural production structure; *i* is the number of sectors (generally including agriculture, forestry, animal husbandry and fishery); *x*_*i*_ is the cumulative percentage of output; *yi* is the cumulative percentage of input.

#### Measuring the impact of RT on G

(1) First, we analyze the relationship between the rationalization index of agricultural production structure and the equality of educational development in the five major agricultural producing areas. In this section, we use the curve estimation to measure the impact of the rationalization index of agricultural production structure on the Gini coefficients in the main agricultural production areas from 2013 to 2018. The education Gini coefficient is used as the *dependent variable*, and the rationalization index of agricultural production structure is used as the *independent variable*. Curve estimation in 11 functional forms is performed, and the estimated coefficient of the function with the smallest *p*-value is selected.
(2) Second, taking Xinjiang as a typical representative, we conduct an in-depth analysis of China's main cotton-producing areas. Towns are located between urban and rural areas and are important hubs connecting urban and rural areas. As a result, it is common for rural populations to move into cities and towns for education. Likewise, it is common for urban populations to move into cities for education. In order to make the analysis results clearer, it is necessary to eliminate the effect of urban population education Gini coefficient on urban, rural and overall. Therefore, when analyzing the situation in Xinjiang separately, we conducted a partial correlation analysis between the agricultural rationalization production index and the education Gini coefficient of urban, towns and rural areas.

## Empirical analysis and its results

### Equality analysis of education development in China's major agricultural producing areas

According to formulas (1) and (2), the average years of education and education Gini coefficient of each province are calculated. Table 1 contains the results and national averages for the five major agricultural producing areas. From the results in Table 1, the average years of education showed an obvious upward trend, and the educational Gini coefficient showed a slow downward trend. For the provinces of China's agricultural main producing areas, it does not show a very obvious lag, and the average education years of some agricultural main producing areas are higher than those of some non-agricultural main producing areas. This is the case in Heilongjiang and Xinjiang, whose average years of education in the past 20 years are 8.996 and 8.838, respectively, ranking 8th and 10th among 31 provinces in China (from large to small). The same is true for the Gini coefficient of provinces in China. The Gini coefficient of some provinces in the main agricultural producing areas is lower than that of non-agricultural main producing areas. The typical province is Heilongjiang, whose average Gini coefficient in the past 20 years is 0.1931, ranking fourth (from small to large).

**Table 1:** Table of average years of education and Gini coefficient in some major agricultural producing areas of China.

**Year**	**Guangxi**		**Henan**		**Heilongjiang**		**Shandong**		**Xinjiang**		**China**	
	**μ**	***G***	**μ**	***G***	**μ**	***G***	**μ**	***G***	**μ**	***G***	**μ**	***G***
2002	7.62	0.2274	8.08	0.211	8.30	0.2052	8.08	0.2411	8.37	0.2487	7.73	0.2457
2003	7.77	0.227	7.97	0.2076	8.41	0.1937	7.85	0.2592	8.38	0.2371	7.91	0.2436
2004	8.02	0.2209	8.22	0.2043	8.49	0.1829	7.94	0.2507	8.49	0.2354	8.01	0.2385
2005	7.66	0.2229	7.99	0.2184	8.46	0.2096	7.72	0.2543	8.20	0.2434	7.83	0.2488
2006	8.03	0.2052	8.05	0.2106	8.53	0.1984	8.09	0.2323	8.30	0.2283	8.04	0.2374
2007	8.03	0.196	8.18	0.2037	8.70	0.1904	8.23	0.2215	8.51	0.2116	8.19	0.2304
2008	7.98	0.1909	8.34	0.2013	8.70	0.1866	8.28	0.2178	8.56	0.2157	8.27	0.2255
2009	8.10	0.1928	8.39	0.2007	8.75	0.1893	8.31	0.2156	8.66	0.2049	8.38	0.2228
2010	8.44	0.1935	8.66	0.1987	9.16	0.181	8.76	0.2152	8.92	0.2061	8.81	0.2108
2011	8.61	0.2092	8.70	0.2054	9.11	0.1818	8.67	0.2217	9.18	0.218	8.85	0.2157
2012	8.42	0.195	8.66	0.1956	9.21	0.1856	8.78	0.2209	9.05	0.2157	8.94	0.2149
2013	8.59	0.1979	8.78	0.2021	9.48	0.1871	8.92	0.2133	8.99	0.2154	9.05	0.2138
2014	8.75	0.2004	9.00	0.2084	9.35	0.1935	8.98	0.2162	9.18	0.2117	9.04	0.2179
2015	8.68	0.2109	8.83	0.2109	9.38	0.1972	9.03	0.2303	9.09	0.2276	9.13	0.226
2016	8.76	0.1996	8.81	0.2065	9.37	0.2048	9.03	0.2293	9.10	0.221	9.13	0.2241
2017	8.76	0.1967	8.94	0.2054	9.42	0.1973	9.12	0.228	9.56	0.2232	9.27	0.2224
2018	8.74	0.1942	8.97	0.2058	9.54	0.1943	9.00	0.2355	9.41	0.2296	9.26	0.224
2019	9.02	0.1967	9.12	0.2097	9.57	0.1972	9.01	0.241	9.17	0.2262	9.33	0.2228

**Figure 2:**
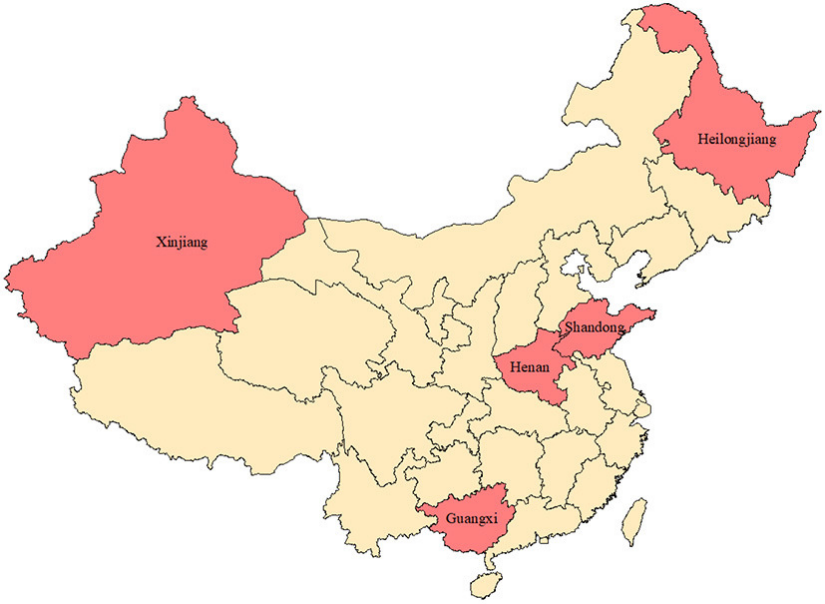
Geographical location of major agricultural producing areas.

Further, an in-depth analysis of five representative agricultural main producing areas is carried out. These provinces are China's food production functional areas and important agricultural production reserves, and the region ranks first in China in the production of one or more agricultural products. Guangxi is an important sugar cane production area, and its sugar cane planting area accounts for more than 60% of China. Henan is an important grain production function area, and its wheat planting area and total output rank first in China. Heilongjiang is an important soybean production reserve, its soybean production accounts for more than 50% of China. Shandong is an important agricultural main producing area. Its grain, meat, aquatic products, fruits, vegetables, and other agricultural products have been stable in the forefront of the country, and the gross agricultural product has always been in the first place in China. Xinjiang is the main cotton production reserve, concentrating more than 80% of China's cotton production. The geographic locations of the five major agricultural producing areas are shown in Figure 2.

In the past 20 years, the development of education in China's major agricultural producing areas has made remarkable achievements. Although there are gaps compared with other regions, the trend of narrowing the gap is very obvious. From the selected five provinces, the characteristics of education development in agricultural main producing areas are mainly reflected in two aspects. On the one hand, compared with themselves, the average education years of residents in the sample provinces are increasing. As can be seen from Table 1, the average years of education in the five sample provinces have increased significantly over the past 20 years, with an average increase of 1.31 years. On the other hand, compared with other provinces, the education development gap of provinces in major agricultural producing areas is also obvious. Compared with the national level, the average years of education in the four provinces except Heilongjiang are lower than the national average. Guangxi's education development performance is the worst. In the past 20 years, its average years of education are lower than the national average.

There are significant differences in the educational development of the provinces in the main agricultural producing areas. From the selected five sample provinces, in the past 20 years, the average educational Gini coefficient of Heilongjiang is 0.193, ranking fourth in the country. The average educational Gini coefficients of Guangxi and Henan are 0.204 and 0.206, ranking 10th and 11th in China, respectively. The average Gini coefficient of education in Xinjiang is 0.223, ranking 15th in the country. Shandong's education Gini coefficient is 0.23, ranking 19th in the country. From the above data, it can be seen that there are significant differences in the equality of education development in the same major agricultural producing areas. Although the GDP of Shandong Province ranks first in these provinces, its Gini coefficient of education is the largest, and the inequality of education development is also the largest. Further analysis shows that among the population aged 6 and over in Shandong, the proportion of the population without schooling is relatively high, and the average proportion in the past 20 years is 8% (6.38% in Henan, 4.98% in Xinjiang, 5.38% in Guangxi and 4% in Heilongjiang). Due to the large base of the population without schooling, the inequality of education development in Shandong is improved, and the Gini coefficient is also large.

### The impact of agricultural production structure changes on the equality of education development in China's major agricultural producing areas

The results of the rationalization index of agricultural production structure in the main agricultural production areas are shown in Table 2. From the results in Table 2, the rationalization index of agricultural production structure in five typical agricultural main producing areas all shows a downward trend. This is closely related to the delineation of grain production functional areas and important agricultural production protection areas. As a result, the development of agriculture, forestry, animal husbandry and fishery in these provinces mainly focuses on one or a few agricultural products.

**Table 2:** Rationalization index of agricultural production structure in major agricultural producing areas of China since 2013.

**Year**	**Xinjiang**	**Heilongjiang**	**Shandong**	**Henan**	**Guangxi**
2013	0.938690027	0.883110024	0.912870486	0.975728906	0.8844908
2014	0.962229899	0.899181992	0.917542565	0.976742775	0.900423852
2015	0.958174879	0.866949001	0.91154215	0.973939613	0.876962664
2016	0.957887176	0.867338673	0.902407875	0.972094535	0.876879427
2017	0.931276589	0.891768199	0.896519589	0.978152863	0.883452235
2018	0.929767974	0.888435383	0.904152396	0.991096162	0.889256141

Table 3 shows the effect of the agricultural rationalization production index on the education Gini coefficient. It can be seen from Table 3 that the estimated coefficients of the selected five sample provinces are all positive. Although the parameter estimation of Heilongjiang, Shandong and Henan provinces did not pass the test of dominance, the reason for their non-dominance is mainly due to the small amount of data. From these parameters, we can boldly make some guesses: In the main agricultural production areas, there is a positive correlation between the rationalization coefficient of agricultural production structure and the educational Gini coefficient. To verify this relationship, this paper selects Xinjiang as a typical case for in-depth analysis, because its agricultural production mainly focuses on a few kinds of agricultural products such as cotton, beef and mutton.

**Table 3:** Correlation analysis between agricultural production structure and educational development equality in main agricultural production areas.

**Index**	**Xinjiang**	**Heilongjiang**	**Shandong**	**Henan**	**Guangxi**
Model selection	Curve estimation: composite	Curve estimation: composite	Curve estimation: inverse	Curve estimation: power	Curve estimation: linear
Coefficient	0.388	0.422	0.436	0.786	0.497
*p*-Value	0.012	0.185	0.317	0.311	0.09

### In-depth analysis of typical agricultural production province Xinjiang

#### Education development in Xinjiang

(1) The average years of education of the urban and rural in Xinjiang

Xinjiang is the main cotton-producing area in China. The calculation of the average years of education in Xinjiang can intuitively reflect the basic situation of education development in Xinjiang in recent years. In this section, we calculate and compare the average years of education of the urban and rural populations in Xinjiang over the past 20 years.

According to the calculation results in Table 4, it can be found that:

**Table 4:** Basic situation of education development in Xinjiang since 2001.

**Year**	**N_0_**	**R_1_**	**R_2_**	**R_3_**	**R_4_**	**R_5_**	**MY**	**MY_1_**	**MY_2_**	**MY_3_**
2001	16,808,291	9.10%	41.63%	30.26%	13.37%	5.63%	7.73	10.2082	9.2190	6.8779
2002	18,221	7.74%	35.80%	31.73%	14.85%	9.88%	8.37	10.3813	10.2772	6.9048
2003	17,269	6.57%	36.79%	34.28%	12.36%	9.99%	8.38	8.8515	8.4419	7.1100
2004	17,515	6.62%	34.32%	35.22%	13.94%	9.89%	8.49	10.3721	9.0207	7.1954
2005	243,918	8.08%	34.94%	36.03%	12.20%	8.75%	8.20	9.9827	8.9400	7.3384
2006	17,229	6.44%	35.91%	37.51%	11.45%	8.69%	8.30	10.2379	8.5456	7.3982
2007	17,462	4.59%	35.21%	39.45%	11.78%	8.97%	8.51	10.4075	8.4213	7.5983
2008	17,510	4.85%	34.49%	39.37%	11.59%	9.70%	8.56	10.4864	8.2425	7.6071
2009	17,442	3.89%	33.68%	41.16%	11.76%	9.51%	8.66	10.4297	8.7947	7.7426
2010	17,321	3.92%	31.82%	39.25%	13.20%	11.81%	8.92	10.6833	9.6574	7.8203
2011	17,200	3.95%	29.92%	37.32%	14.66%	14.15%	9.18	11.1900	10.7425	7.5949
2012	16,910	3.72%	31.71%	37.95%	13.18%	13.44%	9.05	10.6475	11.1164	7.6612
2013	16,917	4.10%	30.77%	39.59%	12.69%	12.85%	8.99	10.4194	9.6000	7.9256
2014	17,081	3.90%	28.10%	39.84%	14.92%	13.25%	9.18	10.8559	9.2751	8.0059
2015	329,900	4.94%	29.81%	36.86%	13.84%	14.55%	9.09	10.8250	9.6486	7.9127
2016	18,089	4.37%	30.16%	37.28%	14.47%	13.73%	9.10	10.7943	9.8964	7.7850
2017	18,081	3.64%	28.69%	33.70%	15.89%	18.09%	9.55	11.1836	11.1896	7.9303
2018	18,376	4.54%	28.79%	33.76%	15.69%	17.23%	9.40	11.2890	10.0399	7.9699
2019	17,832	4.54%	30.28%	35.42%	14.88%	14.88%	9.17	10.9468	9.0665	8.1152
2020	24,021,805	3.70%	30.33%	33.96%	14.21%	17.80%	9.43			

N0, Population aged 6 and above; R1, the proportion of the population without schooling; R2, the proportion of the population with primary school; R3, the proportion of the population with junior middle school; R4, the proportion of the population with high school and secondary school; R5, the proportion of the population with college or above; MY, the average education years; MY1, the average education years of the urban population; MY2, the average education years of the town population; MY3, the average education years of the rural population.

Firstly, in the past 20 years, the MY in Xinjiang has been increasing, and the population distribution structure at all levels of education has been gradually optimized. Taking the three censuses (2001, 2010, and 2020) as the boundary, it can be seen from Table 4 that the MY of Xinjiang's population increased from 7.73 in 2001 to 9.43 in 2020, and Per capita years of education increased by 1.7 years. Some years, although not census data but sample data, but also can reflect the improvement of Xinjiang MY from the side. Compared with the average years of education in China, the average years of education in Xinjiang from 2002 to 2012 were higher than the average level of China. From 2013 to 2019, the average years of education in four years were lower than the average level of China, which were 2013, 2015, 2016, and 2019. But in terms of specific numbers, Xinjiang and China are very close, which can also show that the average growth rate of years of education in agricultural main producing areas is lower than that in China.

Secondly, from the perspective of population distribution structure at all levels of education. Among the population aged 6 and over, the proportion of junior middle school population is the highest, followed by the proportion of primary school population. Benefiting from the full implementation of China's nine-year compulsory education from 2006 to 2008, it can be found that the proportion of junior high school and above education population has increased rapidly in the following years. The junior high school population reached a maximum of 41.16% in 2009 after the full implementation of compulsory education. The proportion of people above senior high school also reached its peak in 2017, 15.89% and 18.09% respectively.

Thirdly, from the perspective of urban-rural differences, the population of MY1 and MY2 in Xinjiang is significantly higher than that of MY3. MY1 in Xinjiang is perennially more than 10 years, reaching 11.28 in 2018. MY2 in Xinjiang maintained at more than 8 years, some years at more than 11 years, reached 11.19 in 2017. Xinjiang's MY3 was significantly lower, reaching 8.12 only in 2019. The remaining years are under 8 years, there is still a certain gap from the 9-year compulsory education goal. From the calculated growth rate (In the calculation process, the average data from 2016 to 2019 are regarded as the last year data, and the average data from 2001 to 2003 are regarded as the first year data, in order to avoid the calculated average annual growth rate cannot reflect the development trend of the average years of education.), the average annual growth rate of MY1 in Xinjiang is 0.704%, the average annual growth rate of MY2 is 0.448%, and the average annual growth rate of MY3 is 0.777%. From the above calculation process, it can be seen that the average annual growth rate of MY3 and MY1 in Xinjiang is very close. In the case of a large base gap, this trend will make the education gap between urban and rural areas continue to expand.

(2) Gini coefficients of urban and rural areas in Xinjiang

To better analyze the inequality of education development in Xinjiang, in this section, we use the education population data of cities, towns and rural areas to calculate education Gini coefficients in Xinjiang. At the same time, the educational Gini coefficient of the whole population in Xinjiang is decomposed in urban and rural areas to explore the causes of inequality in educational development. The results are shown in Table 5.

**Table 5:** Urban-rural decomposition of educational inequality in Xinjiang.

**Year**	**G_3_**	**G_2_**	**G_1_**	**G_0_**	**Urban contribution**	**Rural contribution**	**Intergroup difference contribution**
2001	0.2177	0.2474	0.2209	0.2486	0.1361	0.3422	0.5217
2002	0.2232	0.2114	0.2123	0.2487	0.1211	0.3243	0.5546
2003	0.2032	0.2329	0.1888	0.2371	0.1044	0.3132	0.5824
2004	0.2059	0.2268	0.2122	0.2354	0.1362	0.3119	0.5519
2005	0.2235	0.2403	0.2334	0.2434	0.1584	0.3244	0.5172
2006	0.1986	0.2439	0.2211	0.2283	0.1722	0.2987	0.5292
2007	0.1762	0.2143	0.2131	0.2116	0.1863	0.2752	0.5385
2008	0.1734	0.2059	0.2223	0.2157	0.1944	0.2603	0.5454
2009	0.1682	0.2088	0.2106	0.2049	0.1931	0.2655	0.5414
2010	0.1710	0.2100	0.2006	0.2124	0.2078	0.2292	0.5630
2011	0.1872	0.1989	0.1860	0.2180	0.1986	0.2265	0.5750
2012	0.1861	0.2018	0.1965	0.2157	0.2104	0.2293	0.5603
2013	0.1949	0.2313	0.2014	0.2154	0.2179	0.2460	0.5361
2014	0.1828	0.2215	0.1974	0.2118	0.2359	0.2190	0.5451
2015	0.2067	0.2242	0.2087	0.2276	0.2431	0.2199	0.5370
2016	0.1941	0.2173	0.2034	0.2209	0.2554	0.2007	0.5439
2017	0.1987	0.1978	0.1964	0.2232	0.2519	0.1895	0.5587
2018	0.2109	0.2290	0.1939	0.2296	0.2719	0.1876	0.5405
2019	0.2053	0.2388	0.2022	0.2262	0.2940	0.1859	0.5201

G0 is Gini coefficient of education in Xinjiang; G1 is Gini coefficient of Urban Education in Xinjiang; G2 is Gini coefficient of Town Education in Xinjiang; G3 is Gini coefficient of rural education in Xinjiang; RT0 is the Rationalization Index of Agricultural Production Structure in Xinjiang.

It can be seen from Table 5 that the Gini coefficient of rural education in Xinjiang is lower than that in urban areas in most years. Combined with the above analysis, it can be found that the overall development of rural education in Xinjiang lags behind, but its internal gap is lower than that of cities and towns. Taking 2009 as the dividing point, the Gini coefficient of rural population education before 2009 showed a significant downward trend, with a decline rate of 22.73% between 2001 and 2009, and a significant upward trend after 2009, with a rise rate of 22.06% between 2009 and 2019. The overall proportion of rise and decline was similar, but the base was different, and the final result was an overall downward trend. For the Gini coefficient of population education in Xinjiang towns, the overall trend is the same as that in rural areas, except that the proportion of decline between 2001 and 2009 (15.60%) and increase between 2009 and 2019 (14.39%) is lower than that of rural population. For the Gini coefficient of urban population education in Xinjiang, taking 2011 as the dividing point, the Gini coefficient decreased by 15.79% from 2001 to 2011, increased by 8.71% from 2011 to 2019, and the Gini coefficient of urban population education in Xinjiang was 0.2022 in 2019. Among them, the value of the Gini coefficient of urban population education in Xinjiang was the smallest and the inequality of education development was the lowest.

From the above development trend can be seen, Xinjiang urban and rural education development inequality is objective existence. Combined with the previous analysis of the average years of education of various parts of the population in Xinjiang, it can also be found that there is a large gap between urban and rural education development in Xinjiang. However, it is impossible to obtain a more intuitive analysis from the Gini coefficients of cities, towns and villages, especially when their Gini coefficients are relatively close. Therefore, further research decomposes the Gini coefficient of overall population education in urban and rural areas to analyze the impact of urban-rural differences on education inequality in Xinjiang, the main agricultural producing area.

Additionally, from Table 5, we can also find that urban-rural differences are the most critical factors affecting the inequality of education development in Xinjiang. On the whole, the urban contribution of education Gini coefficient in Xinjiang is increasing, from 13.61% in 2001 to 29.40% in 2019. The rural contribution of the Gini coefficient of education in Xinjiang is declining, from 34.22% in 2001 to 18.59% in 2019. However, its contribution to urban-rural differences is always above 50%, with a maximum value of 58.24% in 2003 and a minimum value of 51.72% in 2005. From the data of the past 20 years, the contribution of urban-rural differences to the inequality of education development in Xinjiang is always greater than the sum of urban and rural contributions. Therefore, it can be considered that urban-rural difference is the most critical factor affecting the inequality of education development in Xinjiang. The microdata from China Education Panel Survey (CEPS; 2014–2015 follow-up data) can also confirm this view. By analyzing the follow-up data of CEPS from 2014 to 2015, this paper compares the main school expenditures of agricultural and non-agricultural households in the compulsory education stage (free compulsory education in China). It can be found from Table 6 that the main school expenditures of agricultural households in one semester are about 30% higher than those of non-agricultural households. Correspondingly, the proportion of households whose economic conditions are “Somewhat poor” and “Very poor” is 22.93 and 5.69%, and the proportion of non-agricultural accounts was 9.9 and 1.61%. It can be seen that the proportion of “Somewhat poor” and “Very poor” in agricultural households is much higher than that in non-agricultural households. Therefore, under the condition of high education expenditure and low income in agricultural households, the gap between urban and rural education is more obvious.

**Table 6:** Comparison of economic conditions and main school expenditures between agricultural and non-agricultural households.

**Type**	**Family economic condition**					**Main campus expenditure for a semester (Unit: Yuan)**						
	**Moderate**	**Somewhat poor**	**Somewhat rich**	**Very poor**	**Very rich**	**Book fees**	**Textbook tutoring fees**	**School uniform fee**	**Activity fee**	**Insurance premium**	**Meal cost**	**Total**
Agricultural Hukou	3,250	1,108	191	275	9	123	128	187	110	133	775	1,456
Non-agricultural Hukou	2,096	258	195	42	16	86	53	129	94	111	540	1,013

At the same time, Xinjiang is also a major agricultural province, and its cotton and mutton have absolute advantages in the country. As the main cotton-producing area in China, its agricultural development has an important impact on urban-rural differences, and urban-rural differences are the most critical factor in the formation of educational development inequality. Therefore, further research focuses on the impact of agricultural production structure on the inequality of education development in Xinjiang.

#### Changes of agricultural rationalization index in Xinjiang

The rationalization index of agricultural production in Xinjiang from 2000 to 2018 calculated according to formula (5) is shown in Table 7.

**Table 7:** Rationalization index of agricultural production structure in Xinjiang.

**Year**	**RT_0_**	**Year**	**RT_0_**
2000	0.987	2010	0.913
2001	0.978	2011	0.972
2002	0.973	2012	0.959
2003	0.956	2013	0.939
2004	0.941	2014	0.962
2005	0.894	2015	0.958
2006	0.919	2016	0.957
2007	0.899	2017	0.931
2008	0.913	2018	0.929
2009	0.922		

It can be seen from Table 7 that RT_0_ showed an overall downward trend from 2000 to 2018. From the previous theoretical analysis, the decline of RT_0_ is related to the gradual concentration of crop production in cotton production and livestock products in beef and mutton production in the development process of Xinjiang. With the improvement of the centralized production efficiency of major agricultural products such as cotton and beef and mutton, agricultural development is increasingly concentrated on several major agricultural products. As a result, the production structure of agriculture, forestry, animal husbandry and fishery is unbalanced and the RT_0_ is reduced.

Moreover, with the improvement of the centralized production efficiency of major agricultural products such as cotton and beef and mutton, agricultural development is increasingly concentrated on a few agricultural products such as cotton and beef and mutton. This also led to agricultural, forestry, animal husbandry and fishery production structure imbalance, agricultural production structure rationalization index is thus reduced.

#### Influence of agricultural production structure change on inequality of education development in Xinjiang

The inequality of education development in Xinjiang needs to comprehensively consider multiple factors. The difference in education between urban and rural areas cannot be separated from the rapid advancement of urbanization. Each advancement of urbanization will significantly change the agricultural production structure, especially in Xinjiang. Therefore, the impact of agricultural production structure changes on the development of education in Xinjiang is a factor that cannot be ignored.

As far as Xinjiang is concerned, agricultural development has the characteristics of large-scale and mechanization. Adjustment and optimization of agricultural production structure is an important way to attract rural population and develop rural economy. But with the advancement of urbanization, rural population gradually shift to the city. And with agriculture, forestry, animal husbandry and fishery concentrated on a few kinds of agricultural products, Xinjiang's agricultural production structure rationalization index showed a downward trend.

To eliminate the effect of urban population education Gini coefficient on urban, rural and overall, RT_0_, G_0_, G_1_, G_2_ and G_3_ are used in a partial correlation analysis. The results are shown in Table 8.

**Table 8:** Partial correlation analysis results.

**Index**	**Coefficient**	**Coefficient**	**Coefficient**
Dependent variable	Agricultural production structure rationalization index		
**Control variable**	**Gini coefficient of Town Education** **in Xinjiang**		
Independent variable	G_0_	G_1_	G_3_
Relevance	0.384	−0.48	0.398
Significance (single-tailed)	0.064	0.026	0.057
Self-service sampling deviation	−0.001	0.025	0.015

Unless otherwise stated, self-help sampling results are based on 1,000 self-help sampling samples.

Through partial correlation analysis, it is found that RT_0_ has a positive correlation with G_0_, and also has a positive correlation with G_3_. The correlation coefficients are 0.384 and 0.398, which are tested by the 10% significance level. The above results show that the decrease of RT0 will increase G and G_3_ and reduce the inequality of education development in Xinjiang. This is related to the centralized development trend of the agricultural production structure in Xinjiang. By analyzing the data of agriculture, forestry, animal husbandry and fishery in Xinjiang in recent 20 years, it is found that the changes of agricultural production structure in Xinjiang mainly focus on agriculture and animal husbandry. Agriculture focuses on cotton production, while animal husbandry focuses on beef and mutton pork production. In the past 20 years, the cotton production area in Xinjiang has accounted for 40% of the total area of crops in Xinjiang, and the pork production of cattle and sheep is also in the forefront of the country. RT_0_ decreased with the concentration of agricultural production, that is to say, the concentration of cotton, beef and mutton pork reduced the rationalization of the agricultural production structure in Xinjiang. However, this trend does not increase the inequality of education in Xinjiang, but reduces the Gini coefficient of education, indicating that the concentration of agricultural production in Xinjiang has a certain role in promoting the reduction of the inequality of education development in Xinjiang. Taking cotton as an example, Xinjiang is designated as a cotton production reserve, and the subsidies for cotton production are stronger than other provinces. Therefore, the economic situation of rural cotton farmers in Xinjiang has been significantly improved. In the case that most farmers in Xinjiang are planting cotton, this improvement is not a unilateral increase in agricultural development, which can directly increase education investment and reduce RT_0_.

RT0 is negatively correlated with G_1_, which means that the decrease of RT_0_ will promote the increase of G_1_. The emergence of this situation is closely related to the imbalance of the agricultural production structure in Xinjiang. In the reality, the imbalance of agricultural production structure in Xinjiang leads to the need to transport more agricultural and sideline products from other regions, which objectively increases the daily living expenditure of urban residents. This increase in expenditure will affect urban residents' expenditure on education, thereby enhancing G_1_.

Whether urban population or rural population, their education Gini coefficient is related to RT. This relationship is very important for analyzing the differences in urban and rural education development. Through the above analysis, it can be found that the reduction of RT in agricultural main producing areas will promote the decline of educational Gini coefficient and reduce the inequality of regional education development. This process is very long. With the improvement of urbanization level, mechanization and scale development of agricultural production will accelerate this process.

## Conclusions and limitations

### Main conclusions

In summary, there is a positive correlation between the rationalization of agricultural production structure and the Gini coefficient of education in Xinjiang. The emergence of this relationship is related to the concentration of agriculture and animal husbandry in Xinjiang. With the transfer of population from rural to urban, agricultural production is becoming more and more concentrated, which is conducive to narrowing the gap between urban and rural education investment. It is also conducive to reducing the inequality of urban and rural education development. However, Xinjiang represents not only a region but also the basic situation of China's main agricultural producing areas. The following basic conclusions can therefore be drawn: The increase of RT in the main agricultural producing areas does not necessarily reduce the imbalance between urban and rural education development. The improvement of agricultural specialization (i.e., the decrease of RT) can effectively reduce the inequality of education development, and narrow the gap between urban and rural education development.

### Limitations

The limitations of the study are mainly the following aspects. First, the conclusion summarizes the general phenomenon of China's agricultural main producing areas, but the development gap between different agricultural main producing areas is large, which is difficult to be eliminated in the analysis. Second, in the analysis of the relationship between the agricultural production structure and the equality of education development in the main agricultural producing areas of China, due to the number of samples and variable selection, some analysis may have some errors. Therefore, the follow-up study needs to further expand the time range and increase the number of samples to enhance the persuasiveness of the conclusion.

## Data availability statement

The original contributions presented in the study are included in the article/supplementary material, further inquiries can be directed to the corresponding author.

## Author contributions

Data and methodology: JW. Writing and analysis: YY. Both authors contributed to the article and approved the submitted version.

## Funding

This work was supported by Chongqing Social Science Fund Project (Skldy202121 and Skldy202003). The funders had no role in study design, data collection and analysis, decision to publish, and preparation of the manuscript.

## Conflict of interest

The authors declare that the research was conducted in the absence of any commercial or financial relationships that could be construed as a potential conflict of interest.

## Publisher's note

All claims expressed in this article are solely those of the authors and do not necessarily represent those of their affiliated organizations, or those of the publisher, the editors and the reviewers. Any product that may be evaluated in this article, or claim that may be made by its manufacturer, is not guaranteed or endorsed by the publisher.
